# Development and validation of a radiomics-based nomogram for predicting a major pathological response to neoadjuvant immunochemotherapy for patients with potentially resectable non-small cell lung cancer

**DOI:** 10.3389/fimmu.2023.1115291

**Published:** 2023-02-16

**Authors:** Chaoyuan Liu, Wei Zhao, Junpeng Xie, Huashan Lin, Xingsheng Hu, Chang Li, Youlan Shang, Yapeng Wang, Yingjia Jiang, Mengge Ding, Muyun Peng, Tian Xu, Ao’ran Hu, Yuda Huang, Yuan Gao, Xianling Liu, Jun Liu, Fang Ma

**Affiliations:** ^1^ Department of Oncology, The Second Xiangya Hospital, Central South University, Changsha, Hunan, China; ^2^ Department of Radiology, The Second Xiangya Hospital, Central South University, Changsha, Hunan, China; ^3^ Clinical Research Center for Medical Imaging in Hunan Province, Changsha, Hunan, China; ^4^ Department of Pharmaceutical Diagnosis, GE Healthcare, Changsha, China; ^5^ Department of Thoracic Surgery, The Second Xiangya Hospital of Central South University, Changsha, Hunan, China; ^6^ Department of Ministry of science and technology, The Second Xiangya Hospital, Central South University, Changsha, Hunan, China; ^7^ Department of Basic Science, College of Chiropractic, Logan University, Chester field, MO, United States; ^8^ Radiology Quality Control Center, Changsha, Hunan, China

**Keywords:** radiomics, nomogram, major pathological response, NSCLC, neoadjuvant immunochemotherapy

## Abstract

**Introduction:**

The treatment response to neoadjuvant immunochemotherapy varies among patients with potentially resectable non-small cell lung cancers (NSCLC) and may have severe immune-related adverse effects. We are currently unable to accurately predict therapeutic response. We aimed to develop a radiomics-based nomogram to predict a major pathological response (MPR) of potentially resectable NSCLC to neoadjuvant immunochemotherapy using pretreatment computed tomography (CT) images and clinical characteristics.

**Methods:**

A total of 89 eligible participants were included and randomly divided into training (N=64) and validation (N=25) sets. Radiomic features were extracted from tumor volumes of interest in pretreatment CT images. Following data dimension reduction, feature selection, and radiomic signature building, a radiomics-clinical combined nomogram was developed using logistic regression analysis.

**Results:**

The radiomics-clinical combined model achieved excellent discriminative performance, with AUCs of 0.84 (95% CI, 0.74-0.93) and 0.81(95% CI, 0.63-0.98) and accuracies of 80% and 80% in the training and validation sets, respectively. Decision curves analysis (DCA) indicated that the radiomics-clinical combined nomogram was clinically valuable.

**Discussion:**

The constructed nomogram was able to predict MPR to neoadjuvant immunochemotherapy with a high degree of accuracy and robustness, suggesting that it is a convenient tool for assisting with the individualized management of patients with potentially resectable NSCLC.

## 1 Introduction

Non-small cell lung cancer (NSCLC) accounts for 80-85% of all lung cancers and is the leading cause of cancer death worldwide (1). About 20-25% of NSCLCs are resectable at the time of diagnosis, including most stage I–IIIa and a small proportion of stage IIIb tumors (2, 3). The 5-year overall survival (OS) rates of resectable lung cancer patients are unsatisfactory (4). Pathological response to neoadjuvant treatment is a potential surrogate for an early clinical endpoint for long-term survival (5–7). Only about 4% of NSCLC patients achieve a pathological complete response (PCR) after neoadjuvant chemotherapy alone (5), which is far from satisfactory. It is well known that no major advances have been made in neoadjuvant treatments for NSCLC over the 25 years prior to the emergence of immunotherapeutic drugs (5). Checkmate 816, the first stage 3 randomized neoadjuvant immunotherapy-based combination study, confirmed that neoadjuvant immunotherapy in combination with chemotherapy is significantly more efficacious than neoadjuvant chemotherapy alone for resectable NSCLC (8). The neoadjuvant immunochemotherapy group was superior to the neoadjuvant chemotherapy group in both MPR (36.9% vs. 8.9%) and median event-free survival (EFS) (31.6 months vs. 20.8 months) (9, 10). Minimally invasive surgery was more common, there were minimal delays in surgery and minimal differences in treatment-related adverse events. Based on the promising results of this trial, combination treatment with neoadjuvant chemotherapy and nivolumab was formally approved by the FDA in March 2022 (10).

Though neoadjuvant immunochemotherapy has greatly succeeded at treating NSCLC, the MPR% of immunochemotherapy is still approximately 36%, which is relatively low (10). Some patients, especially those with stage IIIA/B NSCLC, had a poor response that required timely changes to their treatment regimen. Immunochemotherapy can also lead to severe immune-related adverse effects (irAEs, such as pneumonitis) in some patients. Given this, finding a reliable approach for predicting MPR before administering neoadjuvant immunochemotherapy is critical to maximizing patient benefit, minimizing risks, and recommending personalized perioperative treatment for patients with potentially operable NSCLC. There is presently no reliable parameter or biomarker that can predict the response to neoadjuvant immunochemotherapy. Tumor PD-L1 expression could predict the efficacy of immunotherapy to some extent, but its predictive value is not as good with combination immunochemotherapy vs. mono-immunotherapy (9). Previous studies have shown that patients who achieve PCR have a distinctive peripheral blood immune status (11), and pretreatment tissue TCR repertoire evenness is also associated with PCR (12). Other reports have shown that baseline neutrophil-to-lymphocyte ratio (NLR) can independently predict the pathological response to neoadjuvant immunochemotherapy (13). However, these serological biomarkers are either too expensive or susceptible to various factors such as inflammation or infection and have not been prospectively validated. They are not presently applied clinically. Alternative predictive biomarkers are needed urgently, especially those that can be detected using a non-invasive method.

Medical imaging, such as computed tomography (CT), plays a vital role in the diagnosis, evaluation, and treatment monitoring of NSCLC (14). Medical images possess a significant amount data that cannot be observed intuitively (15). Data mining of medical images to assist with clinical decision-making has emerged as a hot spot in medical research. Radiomics, first proposed by Philippe (16), refers to an emerging data-driven strategy that can extract either a set of predefined engineered features that describe radiographic aspects of shape, intensity, and texture, or alternatively high-level features (i.e. valvelet). Radiomics have been widely studied to aid in disease diagnosis, prognostication, and the prediction of treatment response to facilitate personalized medicine (17). Several previous studies also used radiomics to predict gene mutation status and the response to chemoradiotherapy for various tumors, with promising results (18, 19). However, to the best of our knowledge, no previous studies have evaluated the potential value of radiomics to predict MPR after neoadjuvant immunochemotherapy in NSCLC.

The present study aimed to develop and validate a radiomics-based model that can efficiently predict MPR for potentially resectable NSCLC treated with neoadjuvant immunochemotherapy. We also explored the integration of CT-based radiomics and clinical data into a multidimensional nomogram to predict MPR.

## 2 Materials and methods

**Figure 1:**
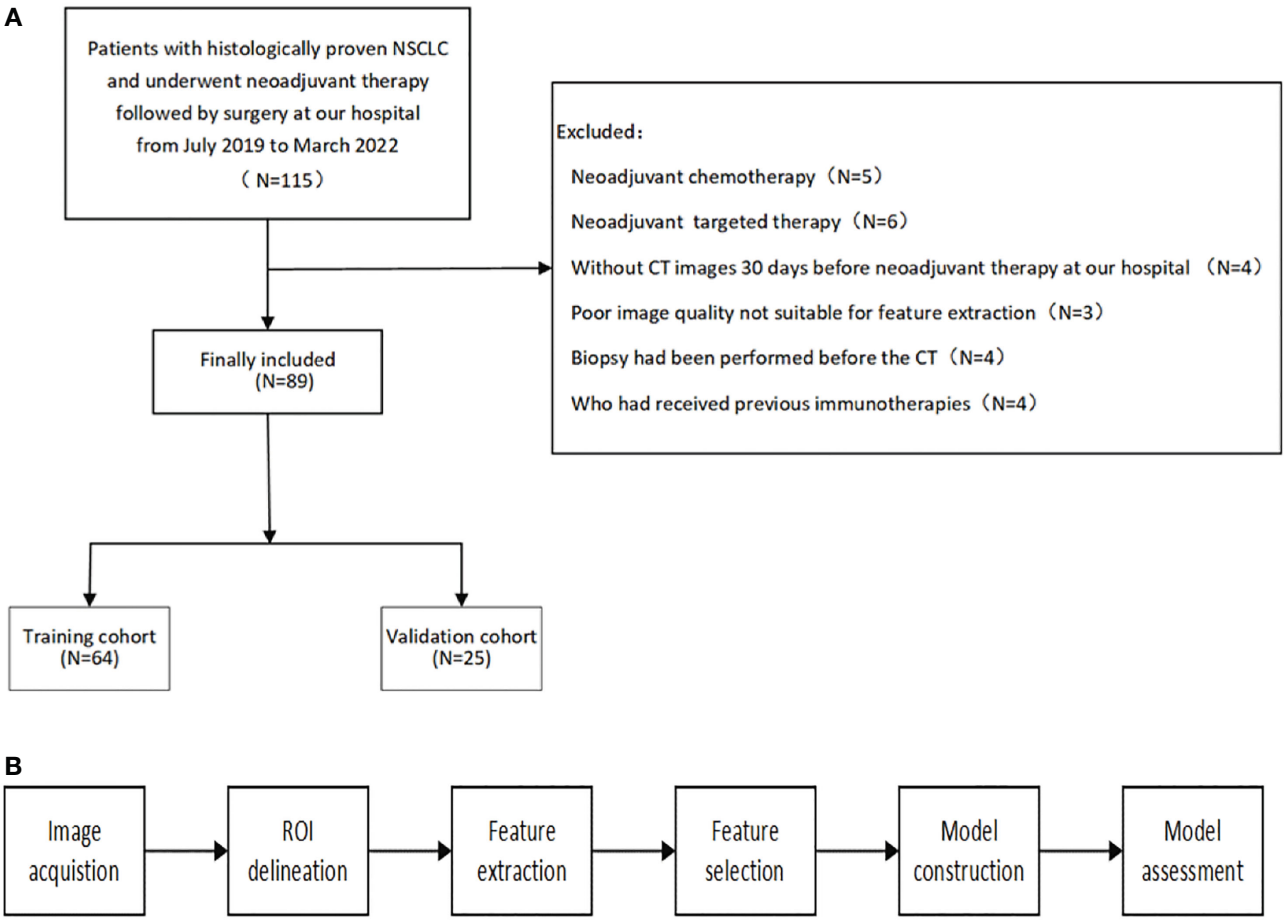
**(A)** patient selection and distribution flowchart. **(B)** model construction and assessment flowchart.

The patient selection and distribution flowchart is shown in **Figure 1A**, and the model construction and assessment flowchart is shown in **Figure 1B**.

### 2.1 Patients

We collected data retrospectively from July 2019 to March 2022. We included all NSCLC patients at our hospital who were: (1) age 18 years or older, (2) pathologically confirmed, (3) clinical stage IB–IIIC, and (4) underwent neoadjuvant immunochemotherapy followed by surgery. The exclusion criteria were as follows: (1) previous immunotherapy, (2) no available pretreatment CT scans, (3) non-measurable lesions per the Response Evaluation Criteria in Solid Tumors (RECIST) 1.1, (4) poor image quality that was not suitable for feature extraction, and (5) a biopsy was performed prior to the available CT. Patient clinicopathological information, including age, sex, stage, PD-L1 expression (Dako 22C3), and details about their neoadjuvant treatment, including agents and course of treatment, were collected from the electronic database. MPR was defined as 0-10% of viable tumor cells remaining in the residual tumor.

### 2.2 Treatment groups

All patients underwent a standard preoperative staging workup that included a pretreatment tumor biopsy, a contrast-enhanced CT scan of the chest and abdomen, contrast-enhanced brain magnetic resonance imaging, and a 99mTc-labeled methylene diphosphonate (99mTc-MDP) whole-body bone scan. Tumors were staged according to the 8th edition of the American Joint Committee on Cancer criteria. The surgeon evaluated all patients to determine whether they were appropriate for surgical intervention or should continue neoadjuvant therapy after two to four cycles of neoadjuvant immunochemotherapy. All pathologic information, including MPR or non-MPR (N-MPR) status, was reconfirmed by a senior pathologist. Patients who achieved MPR or PCR were assigned to the MPR group, and all others were assigned to the N-MPR group.

### 2.3 CT scan protocols

CT scans obtained within 30 days of the treatment start date were analyzed. If there was more than one CT scan, the closest CT scan before the biopsy was used. The included patients underwent CT scans with the following four scanners: Somatom Definition Flash, Siemens, Germany; uCT780, United Imaging, Shanghai, China; Somatom Perspective 128, Siemens, Germany; and Somatom Definition Force, Siemens, Germany. Scanning protocols were as follows: 120 kVp, 100–200 mAs, and pitch 0.75–1.5. Plain CT images with 5-mm thickness cuts were retrieved from the Picture Archiving and Communication System (PACS) for further analysis. CT images were acquired in the supine position at full inspiration for all patients.

### 2.4 Segmentation and radiomic feature extraction

All targeted tumors were manually delineated slice by slice by one author with 5 years of experience with chest CT interpretation using the medical image processing and navigation software 3D Slicer (version 4.10.1, Brigham and Women’s Hospital). Patients with multiple lesions on their CT scan had the lesion with the largest diameter selected. Volumes of interests (VOIs) were then confirmed by another radiologist with 10 years of experience with chest CT interpretation. Images and VOIs were exported in the NII format for further analysis. Prior to radiomic feature extraction, the voxel size of the VOI was resampled to 1*1*1 mm^3^ *via* cubic interpolation to reduce feature value variability due to different voxel sizes. A total of 1746 radiomic features were extracted, including First order, Shape3D, GLCM, GLSZM, GLRLM, NGTDM, GLDM, and high order features.

### 2.5 Reproducibility analysis and feature selection

Given concerns about the reproducibility of radiomic features, we performed an intra-observer reproducibility analysis. One author segmented 50 randomly chosen images at two-time points with an interval time of one month, producing two VOIs of each selected patient. Agreement between feature extractions was assessed using intra-class correlation coefficients (ICCs). An ICC value greater than 0.8 indicated good agreement. To eliminate redundant radiomic features, we performed a two-step feature selection strategy. MRMR was initially used to select the 20 most valuable features. The LASSO method, which is suitable for the regression of high-dimensional data (18, 20), was then used to select the most useful predictive features. A radiomics score (Rad-score) was calculated for each patient *via* the linear combination of selected features weighted by their respective coefficients.

### 2.6 Model construction and validation

We randomly divided the dataset into training and validation sets with a ratio of 7:3. We then constructed the radiomics model, clinical model, and radiomics-clinical combined model. Optimal radiomic features were determined through dimension reduction by MRMR and LASSO. Clinical variables were selected *via* univariate and multivariable logistic regression analysis, which began with the following clinical candidate predictors: age, gender, site, smoking, differentiation, pathological type, cT-stage, cN-stage, immuno-therapy regimen, neoadjuvant cycles, PD-L1 expression, Ki67, and NLR. The retained optimal radiomic features and clinical variables whose P-values were <0.05 were used to develop logistic regression analysis model. AUC, accuracy, sensitivity, specificity, PPV, and NPV were used to evaluate model performance. The DeLong test was used to evaluate differences in the ROCs of various models. Calibration of the training set and validation set of each model was measured using a calibration curve. The Hosmer-Lemeshow test was performed to assess the goodness-of-fit of these models. DCA was also performed on the training set of the three models by calculating the net benefits over a range of threshold probabilities.

### 2.7 Statistical analysis

Statistical analysis was performed using R software (version 3.4.3; http://www.Rproject.org) and IBM SPSS Statistics (version 24; IBM, New York, USA). Quantitative data was compared using Student’s t-test or the Wilcoxon test. Categorical data was compared using the χ2 test. Predictive performance was evaluated with the AUC of the receiver operator characteristic (ROC). The Lasso algorithm was executed using the “glmnet” package, and multivariate binary logistic regression, nomograms, and calibration plots were executed with the “rms” package. Internal validation was performed using the “rms” package. DCA was performed using the “rmda” package. Univariate and multivariate logistic regression analyses were used to select clinical variables with p<0.05 for model construction. A two-sided P value <0.05 was considered statistically significant.

## 3 Results

### 3.1 Clinical characteristics

Eighty-nine eligible patients were included in this retrospective study. The entire cohort was randomly divided into a training set (n = 64) and a validation set (n = 25) at a ratio of 7:3. The demographics and clinicopathological characteristics of the patients in the training and validation cohorts are shown in **Table 1**. The majority of the participants were male smokers aged ≤60 who had squamous cell carcinoma and a PD-L1 expression >1%. Eighty-one patients were male (91.01%) and 8 were female (8.99%). Fifty-four (60.67%) had a smoking history. The majority of the tumor histologies included squamous cell carcinoma (SCC) and adenocarcinoma (ADC). Of these, 41 (64.1%) cases were SCC and 20 (31.2%) were ADC in the training cohort, while 18 (72%) were SCC and 6 (24%) were ADC in the validation cohort. There were no significant differences between the training and the validation sets in terms of clinical characteristics, proving that they could be used as training and validation sets (**Table 1**). MPR was achieved by 62.5% (40/89) of the training cohort and 64% (16/25) of the validation cohort (**Table 1**). No statistical differences in baseline characteristics were identified between patients with and without MPR except for ki-67 (p=0.046) and NLR (p=0.022) in the validation cohort.

**Table 1:** Clinicopathological characteristics of the dataset.

	Training cohort(N=64)				Validation cohort(N=25)				All Patients(%)
	MPR	N- MPR	Total(%)	p-value	MPR	N-MPR	Total(%)	p-value	(n=89)
	(n=40)	(n=24)	(N=64)		(n=16)	(n=9)	(N=25)		
Sex									
**Male**	36	22	58(90.6)		16	7	23(92.0)		81(91.0)
**Female**	4	2	6(9.4)	0.82	0	2	2(8.0)	0.12	8(9.0)
Age(yr)									
**≤60**	23	18	41(64.1)		6	7	13(52.0)		54(60.7)
**>60**	17	6	23(35.9)	0.16	10	2	12(48.0)	0.09	35(39.3)
Smoking History									
**Smoker or ex-smoker**	26	11	37(57.8)		12	5	17(68.0)		54(60.7)
**Never smoker**	14	13	27(42.2)	0.14	4	4	8(32.0)	0.39	35(39.3)
Lung_Lobe									
**Left lung**	13	9	22(34.4)		6	4	10(40.0)		32(36.0)
**Right lung**	27	15	42(65.6)	0.68	10	5	15(60.0)	1	57(64.0)
Differentiation									
**High**	3	1	4(6.2)		2	3	5(20.0)		9(10.1)
**Moderate**	8	6	14(21.9)		7	2	9(36.0)		23(25.8)
**Poor**	23	14	37(57.8)		4	4	8(32.0)		45(50.6)
**Other**	6	3	9(14.1)	0.92	3	0	3(12.0)	0.26	12(13.5)
Pathological_type									
**Squamous carcinoma**	32	9	41(64.1)		13	5	18(72.0)		59(66.3)
**Adenocarcinoma**	7	13	20(31.2)		2	4	6(24.0)		26(29.2)
**Other**	1	2	3(4.7)	0.003	1	0	1(4.0)	0.14	4(4.5)
cT Stage									
**cT1**	6	2	8(12.5)		0	1	1(4.0)		9(10.1)
**cT2**	20	10	30(46.9)		7	4	11(44.0)		41(46.1)
**cT3**	3	8	11(17.2)		3	3	6(24.0)		17(19.1)
**cT4**	11	4	15(23.4)	0.05	6	1	7(28.0)	0.39	22(24.7)
cN_Stage									
**cN0**	3	3	6(9.4)		1	0	1(4.0)		7(7.9)
**cN1**	5	5	10(15.6)		2	3	5(20.0)		15(16.9)
**cN2**	28	15	43(67.2)		11	5	16(64.0)		59(66.3)
**cN3**	4	1	5(7.8)	0.45	2	1	3(12.0)	0.88	8(9.0)
Clinical Stage									
**IIA**	1	2	3(4.7)		0	1	1(4.0)		4(4.5)
**IIB**	2	2	4(6.2)		2	1	3(12.0)		7(7.9)
**IIIA**	23	14	37(57.8)		5	4	9(36.0)		46(51.6)
**IIIB**	12	3	15(23.4)		5	2	7(28.0)		22(24.7)
**IIIC**	2	3	5(7.8)	0.35	4	1	5(20.0)		10(11.2)
Neoadjuvant_Cycle									
**1**	1	2	3(4.7)		0	0	0(0.0)		3(3.4)
**2**	15	9	24(37.5)		8	3	11(44.0)		35(39.3)
**3**	14	5	19(29.7)		5	4	9(36.0)		28(31.5)
**4**	9	6	15(23.4)		3	2	5(20.0)		20(22.4)
**5**	1	2	3(4.7)	0.52	0	0	0(0.0)	0.86	3(3.4)
Immunotherapy_Regimen									
**Pembrolizumab**	16	8	24(37.5)		7	4	11(44.0)		35(39.3)
**Tisleizumab**	9	4	13(20.3)		3	1	4(16.0)		17(19.1)
**Sintilimab**	10	6	16(25)		3	2	5(20.0)		21(23.6)
**Nivolumab**	3	1	4(6.2)		3	1	4(16.0)		8(9.0)
**Toripalimab**	1	1	2(3.2)		0	0	0(0.0)		2(2.2)
**Camrelizumab**	1	4	5(7.8)	0.45	0	1	1(4.0)	0.77	6(6.8)
PD-L1									
**≤1%**	5	5	10(15.6)		1	2	3(12.0)		13(14.6)
**>1%**	29	12	41(64.1)		12	6	18(72.0)		59(66.3)
**Unknown**	6	7	13(20.3)	0.19	3	1	4(16.0)	0.65	17(19.1)
PD-L1									
**≤50%**	23	11	34(53.1)		9	7	16(64.0)		50(56.2)
**>50%**	10	6	16((25)		4	1	5(20.0)		21(23.6)
**Unknown**	7	7	14(21.9)	0.52	3	1	4(16.0)	0.60	18(20.2)
Ki-67									
**≤40%**	9	10	19(29.7)		3	6	9(36.0)		28(31.5)
**>40%**	24	7	31(48.4)		10	2	12(48.0)		43(48.3)
**Unknown**	7	7	14(21.9)	0.06	3	1	4(16.0)	0.05	18(20.2)
NLR									
**≤2.75**	17	10	27((42.2)		2	6	8(32.0)		35(39.3)
**>2.75**	22	14	36(56.2)		13	3	16(64.0)		52(58.4)
**Unknown**	1	0	1(1.6)	0.73	1	0	1(4.0)	0.02	2(2.2)

Data are n/N (%), unless otherwise stated. MPR, Major pathological response.

### 3.2 Predictive model development and testing

#### 3.2.1 Radiomics feature selection and rad-score construction

**Figure 2:**
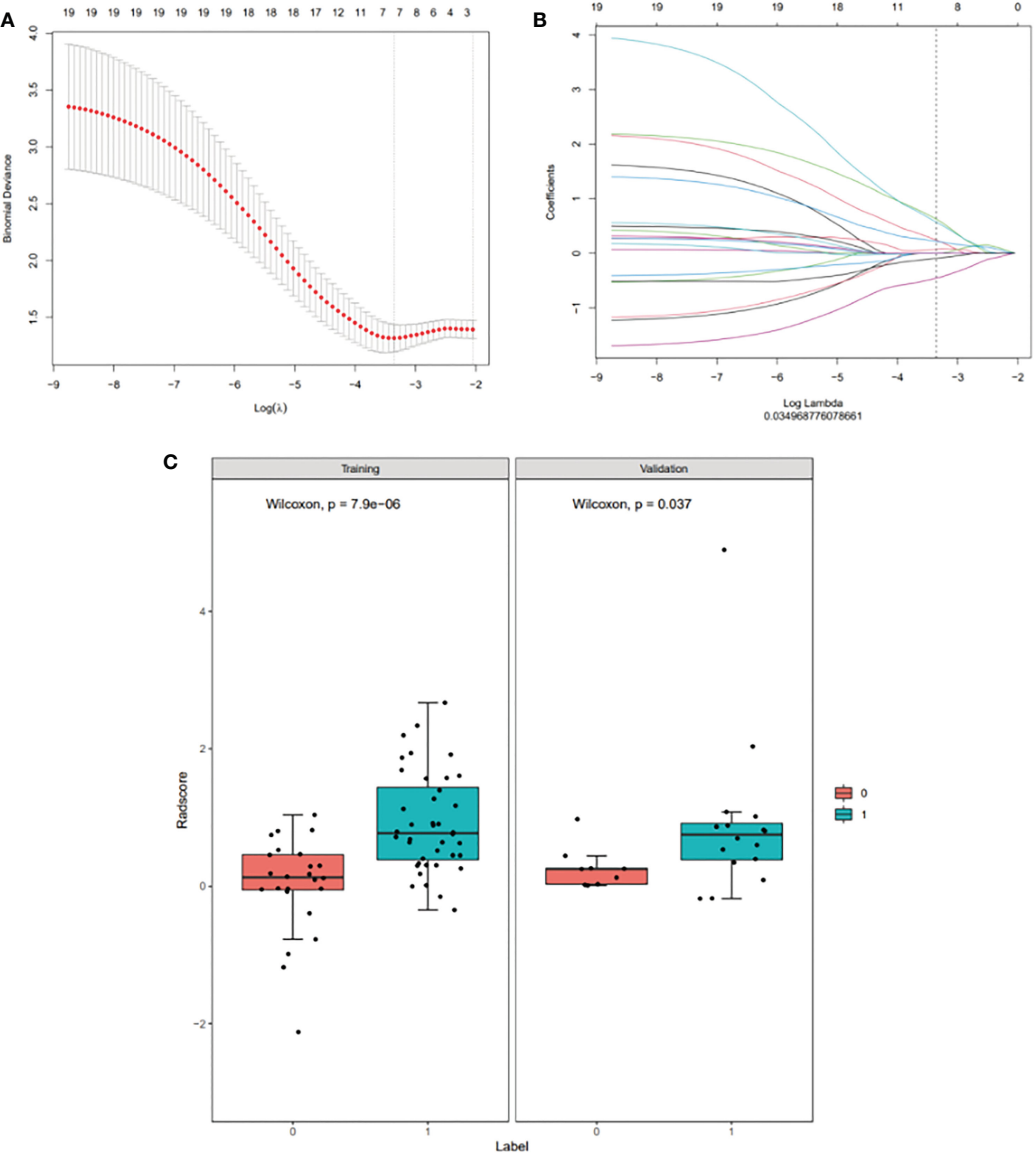
Radiomic feature selection using a least absolute shrinkage and selection operator (LASSO) binary logistic regression model **(A, B)**, with a comparison of the Rad-scores of the MPR and N-MPR groups **(C)**: Red represents the N-MPR group and blue represents the MPR group. There was a significant difference in Rad-score between MPR and N-MPR patients in the training (P<0.001) and validation cohorts (P = 0.037). **(A)** Selection of the tuning parameter (λ) for the LASSO model *via* 10-fold cross-validation based on minimum criteria. The y-axis indicates binomial deviance. The lower x-axis indicates the log (λ). Numbers along the upper x-axis represent the average number of predictors. The optimal λ value of 0.035 with log (λ)= - 3.353 was selected. **(B)** LASSO coefficient profiles (y-axis) of the 20 texture features. The upper and lower x-axis has the same meaning as in Fig. 2A. A black vertical line was drawn at the value selected using 10-fold cross-validation in Fig. 2A. The 7 resulting features with non-zero coefficients are shown in the plot. **(C)** Comparison of Rad-scores between the MPR and N-MPR groups. Red represents the N-MPR group and blue represents the MPR group. There was a significant difference in the Rad-scores of MPR and N-MPR patients in both the training (P<0.001) and validation cohorts (P = 0.037).

A total of 1746 radiomics features were extracted, of which 1251 (71.65%) had an intra-class correlation coefficient of 0.8 or more in the reproducibility analysis. These were chosen for subsequent analysis. After dimension reduction with MRMR, the 20 best features were selected. The Lasso algorithm was used for further dimension reduction (**Figures 2A, B**). Seven optimal radiomics features were chosen to construct the models (**Supplement**). There was a significant difference in Rad-score between MPR and N-MPR patients in the training (P<0.001) and validation cohorts (P = 0.037) (**Figure 2C**).

#### 3.2.2 Model building

**Figure 3:**
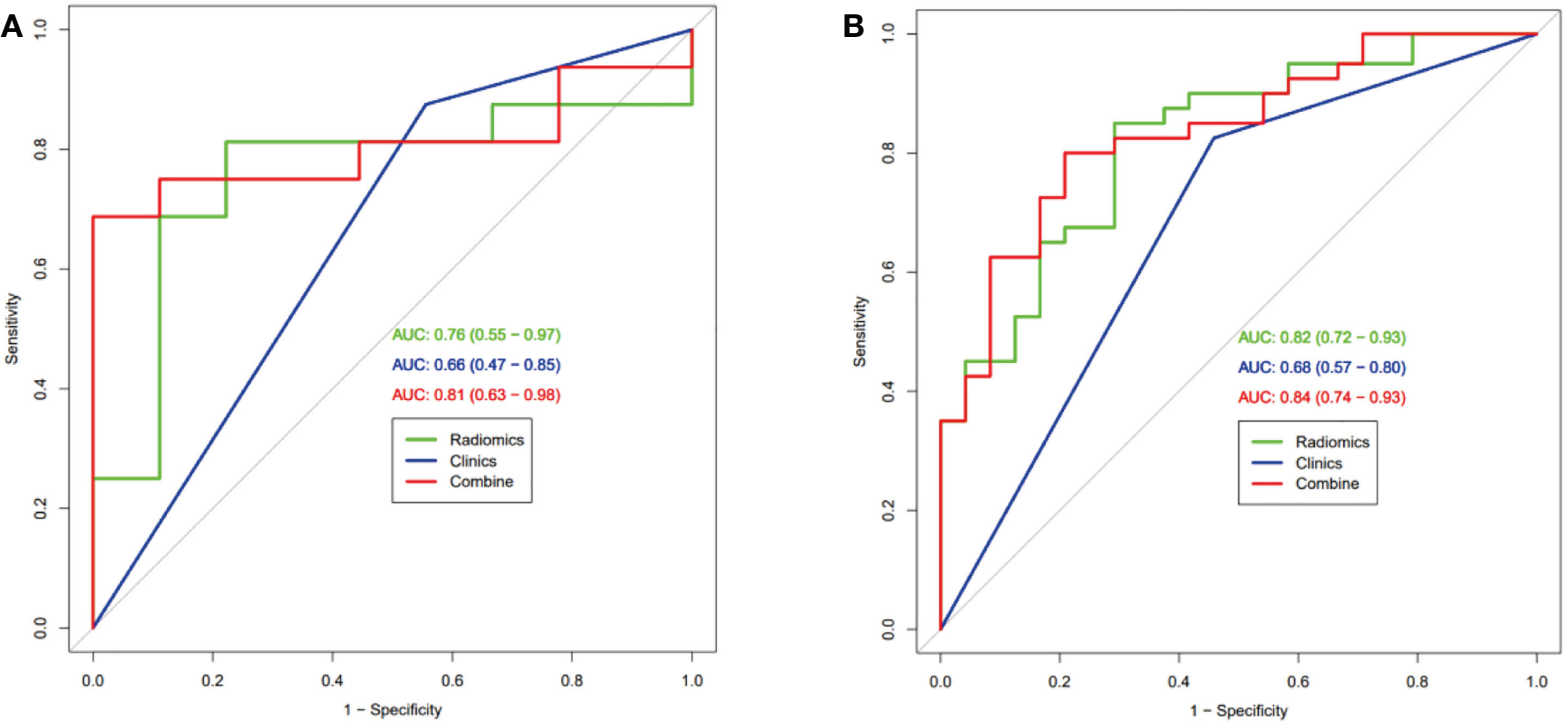
Receiver operating characteristic curve analysis of three models in the training set **(A)** and the validation set **(B)** for predicting Major Pathological Response.

After univariate and multivariate logistic regression analysis, only pathologic type was identified as an independent predictive value and used to build further models (**Table 2**). Three models were built based on selected radiomics features (Rad-score), pathological-type, or their combination (radiomics-only, clinical-only, and radiomics-clinical combined model). The ROC curves of the three models were compared in the training set (**Figure 3A**) and the validation set (**Figure 3B**) for predicting MPR. The AUCs in the training set were 0.82 (95% CI, 0.72-0.93) and 0.68 (95% CI, 0.57-0.80) for the radiomics-only and clinical-only models, respectively. After development in the training cohort, both radiomics-only and clinical-only models accurately predicted MPR in the validation cohort, with AUCs of 0.76 (95% CI, 0.55-0.97) and 0.66(95% CI, 0.47-0.85), respectively. The radiomics-clinical combined model had the best performance, with an AUC of 0.84 (95% CI, 0.74-0.93) and 0.81 (95% CI, 0.63-0.98) in the training and validation sets, respectively.

**Table 2:** Univariate and multivariate analysis of clinical data.

	Univariate analysis		Multivariate analysis	
Variable	OR (95%CI)	P-value	OR (95%CI)	P-value
**Gender**	0.82(0.14-4.84)	0.82	–	
**Age**	1.04(0.97-1.11)	0.25	-	
**Smoking**	2.19(0.78-6.17)	0.14	–	
**Drinking**	1.00(0.31-3.22)	1.00	-	
**Lung Lobe**	1.25(0.43-3.59)	0.68	–	
**Differentiation**	1.09(0.53-2.26)	0.82	-	
**Pathological type**	0.18(0.06-0.56)	0.00	0.18 (0.06; 0.56)	0.0033
**Tumor location**	0.67(0.22-2.02)	0.47	-	
**cT stage**	0.89(0.54-1.49)	0.67	–	
**cN stage**	1.56(0.78-3.13)	0.21	-	
**Immunotherapy regimen**	0.76(0.54-1.07)	0.12	–	
**Neoadjuvant cycle**	0.97(0.58-1.63)	0.92	-	
**PD-L1**	1.35(0.27-6.79)	0.72	–	
**Ki-67**	4.60(0.39-53.74)	0.22	-	
**NLR**	0.79(0.53-1.16)	0.23	–	

The accuracy, sensitivity, specificity, PPV, and NPV of the radiomics-only, clinical-only, and radiomics-clinical combined models in the training and validation cohorts were compared and illustrated in **Table 3**. These quantitative evaluation metrics were balanced between their respective training and validation set in all three models. They were superior in the radiomics-only model compared with the clinical-only model but performed best in the radiomics-clinical combined model. ACC was 79%, 72%, and 80%, sensitivity 85%, 83%, and 80%, specificity 71%, 54%, and 79%, PPV 83%, 75%, and 86%, and NPV 74%, 65%, and 70% for the radiomics-only, clinical-only and combined models in the training set, respectively (**Table 3**). ACC was 80%, 72%, and 80%, sensitivity 81%, 87%, and 75%, specificity 78%, 44%, and 89%, PPV 87%, 74%, and 92%, and NPV 70%, 67%, and 67% for the radiomics-only, clinical-only, and combined models, respectively.

**Table 3:** Model predictive performance.

	Accuracy (95%CI)	Sensitivity	Specificity	PPV	NPV
**R_train**	0.80(0.68-0.89)	0.85	0.71	0.83	0.74
**R_test**	0.80(0.59-0.93)	0.81	0.78	0.87	0.70
**C_train**	0.72(0.59-0.82)	0.83	0.54	0.75	0.65
**C_test**	0.72(0.51-0.88)	0.88	0.44	0.74	0.67
**Combine_train**	0.80(0.68-0.89)	0.80	0.79	0.86	0.70
**Combine_test**	0.80(0.59-0.93)	0.75	0.89	0.92	0.67

Performance comparisons of the radiomics (train cohort + validation cohort), clinical (train cohort+validation cohort) and combined models (train cohort + validation cohort) in terms of ACC, sensitivity, specificity, PPV, and NPV.R_train: training cohort of Radiomics signature, R_test: validation cohort of Radiomics signature, C_train: training cohort of clinical signature, C_test: testing cohort of clinical signature, combine train: training cohort of radiomics-clinical combined signature, combine test: validation cohort of radiomics-clinical combined signature.

ACC, Accuracy; NPV, negative predictive value; PPV, positive predictive value.

#### 3.2.3 Radiomics-based nomogram construction

**Figure 4:**
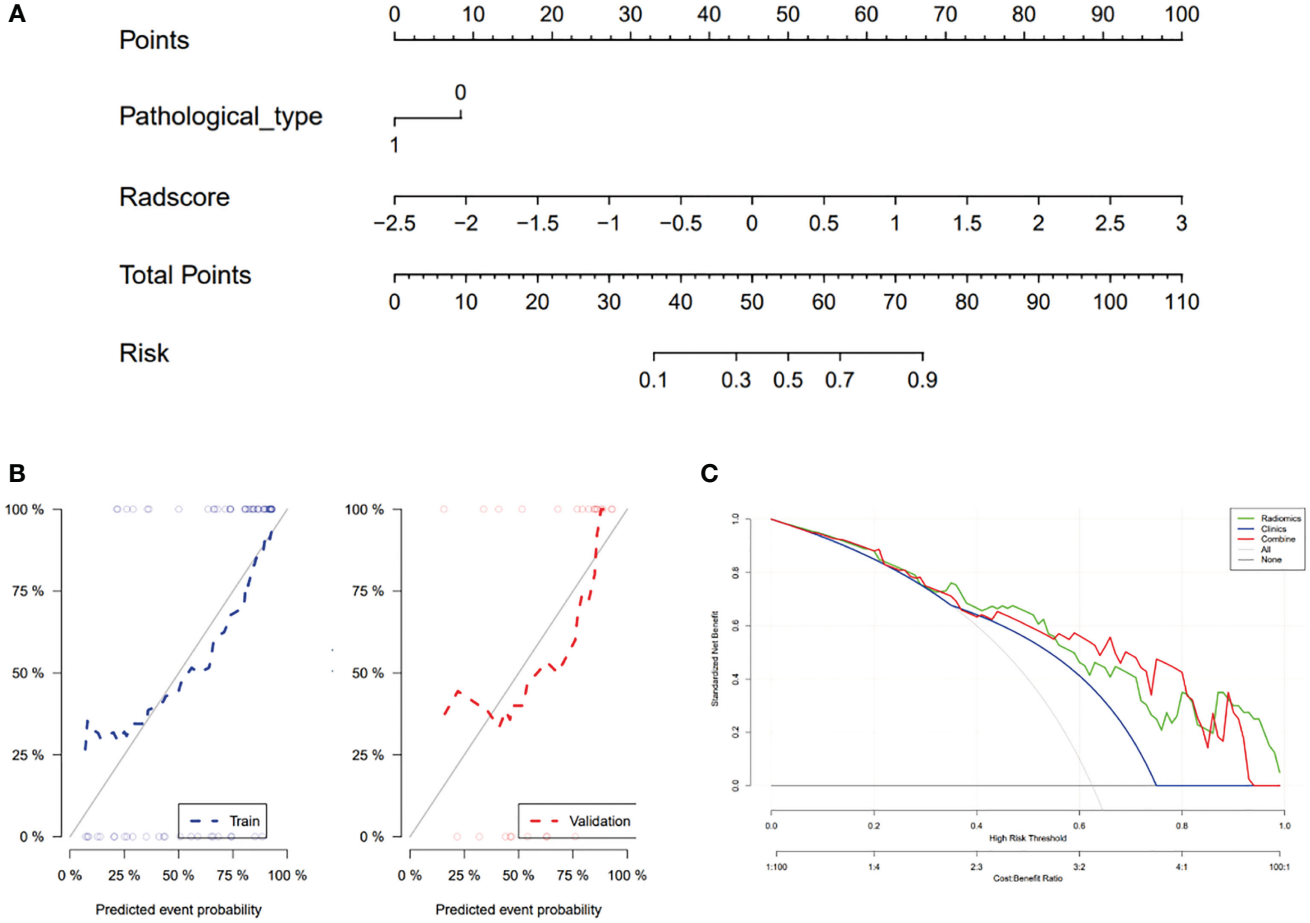
Nomogram, Calibration curve and Decision curve analysis. **(A)** Nomogram and **(B)** Calibration curve for Major Pathological Response in the training and validation groups. **(C)** Decision curve analysis for each model (clinical (Clinics) model, radiomics (Radiomics) model, and integrated (combined) model).

The radiomics-clinical combined model, which incorporated Rad-score and pathologic type, was developed and presented as a nomogram to predict MPR to neoadjuvant immunochemotherapy (**Figure 4A**).

#### 3.2.4 Performance and clinical utility of the constructed nomogram

The calibration curve of the nomogram for the probability of MPR demonstrated good agreement between prediction and observation in the training and validation cohorts of the combined model (**Figure 4B**). The Hosmer-Lemeshow test with a calibration curve had P-values of more than 0.05 (P=0.433, P=0.683 respectively) in the training and test cohorts, suggesting that the nomogram model fit well with the data.

The decision curve analysis (DCA) for the nomogram is presented in **Figure 4C**. The decision curve showed that if the threshold probability of the patient is between 0.3 and 0.95, using both the radiomics model and combined model in the current study to predict MPR added more benefit than a treat-all-patients or treat-none scheme. However, the net benefit of the clinical model was worse than that of the radiomics and combined models.

## 4 Discussion

Accurate treatment response prediction is critical to stratifying and selecting patients who can benefit most from neoadjuvant immunochemotherapy and avoiding ir-sAEs. The present study developed and validated an accurate radiomics-clinical combined model to predict a major pathologic response to neoadjuvant immunochemotherapy in patients with potentially resectable NSCLC, with a favorable AUC (0.84) and a high sensitivity (80%) and specificity (79%), outperforming radiomics-only and clinical-only models.

Pre-treatment CT images show intratumor spatial variation. Previous studies have shown that pre-treatment CT radiomics could reflect tumor CD8 cell infiltration and objective response to immune checkpoint blockade (ICB) monotherapy (21). The combination of immunotherapy and chemotherapy could have a synergistic effect. Several studies have reported on the promising predictive performance of radiomics models in patients with advanced NSCLC receiving immunotherapy or immunochemotherapy. However, this is the first study to the best of our knowledge to predict MPR after neoadjuvant immune-chemotherapy in potentially resectable NSCLC using a radiomics-clinical combined method.

Due to the multiple mechanisms of immunotherapy, atypical patterns of response produced by immunotherapy (e.g., pseudo-progression, delayed responses, and hyper-progression) cannot be correctly assessed using traditional response criteria, making it difficult to judge the benefits of immunotherapy. The pathological test remains the gold standard. MPR is an early surrogate of long-term survival. The nomogram constructed in the present study only contains Rad-score and pathological type, which can be obtained with a routine CT scan and biopsy. The present model can also apply to advanced NSCLC patients to some extent, patients with higher score according to the nomogram are likely to respond better to immunochemotherapy.

Pathological type was the only chosen clinical variable to build the nomogram through univariate and multivariate logistic regression analyses in the present work. Some phase 3 randomized trials, such as Keynote 024 (22), CheckMate 026 (23), CheckMate 227 (24), and CheckMate 017 (25), have already illustrated that squamous NSCLC benefits more from immune checkpoint inhibitors (ICIs) than adenocarcinoma NSCLC. There are two reasons for this: first, both PD-L1 expression and TMB, the two major biomarkers for immunotherapy, are significantly higher in lung squamous carcinoma than lung adenocarcinoma; second, previous studies reported the majority of genomic alterations are distinct between squamous and adenocarcinoma NSCLC since they have different origins (26, 27). The difference in tumor infiltrating lymphocytes (TILs) between the two subtypes may also contribute to ICI clinical outcomes (28). In our study, 66.3% of the patients had squamous carcinoma, which is higher than in CheckMate 816 (50.8%). Our total MPR% (62.9%) thus far exceeded that of CheckMate 816 (36.9%). In addition, the MPR% of both the training and validation sets in the lung squamous carcinoma subgroup was more than 2-fold higher than the lung adenocarcinoma subgroup (**Table 1**).

PD-L1 failed to predict MPR in our study and was not chosen for model construction. Several neoadjuvant immunochemotherapy clinical trials, such as NADIM, LCMC3, and NEOSTAR, have already confirmed that PD-L1 is not an ideal biomarker for predicting MPR (7, 10, 29). In these studies, although a high expression of tumor PD-L1 was associated with MPR, it was not sensitive enough since a substantial fraction of patients with low PD-L1 expression also achieved MPR/PCR. Neither univariate nor multivariate logistic regression analyses in our study showed that PD-L1 is a potential predictor, which is consistent with these findings.

NLR also failed to predict MPR in NSCLC patients receiving neoadjuvant immunochemotherapy in the present work, which is inconsistent with a previous study on the topic (13). In that study, a high baseline NLR level correlated with a poor pathological response. The reasons for this may be as follows: first, the sample sizes in that study (79 patients) and ours (89 patients) were small; second, both studies were retrospective, making bias inevitable; Last but not least, NLR is an inflammatory indicator and is susceptible to various factors, such as bacterial infection, that are common in lung cancer patients. Extensive cohort studies are needed to confirm the value of NLR.

Our nomogram, which was constructed based on Rad-score and pathological type, performed exceptionally well on calibration curve and decision curve analysis, which means that the nomogram fitted well with our data and was useful clinically.

Despite its promising results, our study has several limitations. First, this is a retrospective study, making bias inevitable. A prospective study is needed to further verify our proposed model. Second, our sample size was small and imbalanced. As neoadjuvant immunochemotherapy was recently approved by the FDA in March 2022, the study period was short. Moreover, most participants were male smokers, had squamous carcinoma, and had PD-L1 levels >1%. Third, only pretreatment CT images were able to predict neoadjuvant immunochemotherapy response. Dynamic changes in radiomics (delta radiomics) and other potentially useful clinic-pathological factors, such as tumor infiltrating lymphocyte, gene-mutation profile, PET images, or serum densities of specific subgroups of immune cells has not yet been investigated. Fourth, only the targeted thoracic tumors were delineated manually and analyzed. Accurate automatic segmentation methods should be explored and developed in the future. The radiomics of lymph nodes may also provide valuable information for predicting the response to neoadjuvant immunochemotherapy. Although our current results proved that this model was useful, further confirmation in a larger cohort is mandatory before it can be applied to routine clinical practice.

## 5 Conclusion

A constructed easy-to-use nomogram that incorporates radiomics and clinical feature was able to predict a major pathological response to neoadjuvant immunochemotherapy with high levels of accuracy and robustness. Such a nomogram makes it a convenient tool for assisting with the individualized management of potentially resectable NSCLC.

## Data availability statement

The original contributions presented in the study are included in the article/**Supplementary Material**. Further inquiries can be directed to the corresponding authors.

## Ethics statement

This study was performed in accordance with the ethical standards of the Declaration of Helsinki and was approved by the institutional review board of our institution (Approved Number: 2022-032), which waived the requirement for informed consent for this retrospective study.

## Author contributions

Conceptualization: XL, JL, FM. Methodology: CyL, WZ, HL. Software: CL, YJ, YS. Validation: JX, XH. Formal analysis: HL, JX. Investigation: MP, MD. Resources: TX, YH, and AH. Writing - original draft preparation: CyL, WZ. Writing review and editing: YW, MD, YG. Supervision: XL, JL and FM. All authors contributed to the article and approved the submitted version.
