# Supplementary figures and images for: Development and validation of a radiomics-based nomogram for predicting a major pathological response to neoadjuvant immunochemotherapy for patients with potentially resectable non-small cell lung cancer
Source: Front Immunol. 2023 Feb 16;14:1115291. doi: 10.3389/fimmu.2023.1115291 (PMC9978193; doi:10.3389/fimmu.2023.1115291)

The 7 optimal signatures selected.


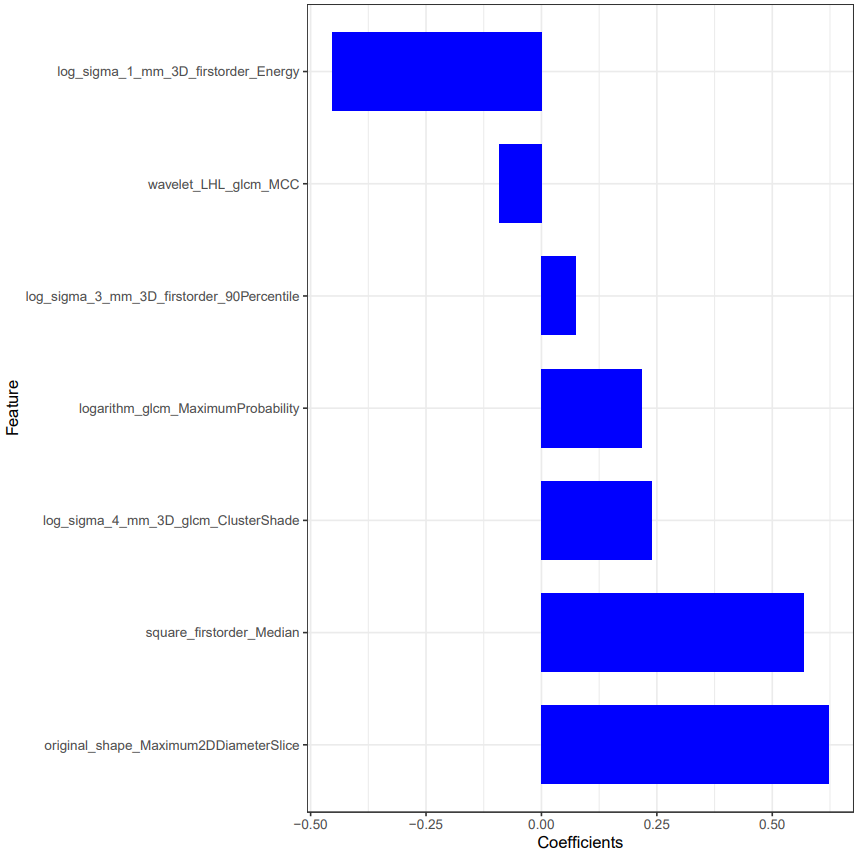

Supplement: Supplementary file 1 [file DataSheet_1.docx]
